# Translation and validation of the Arabic version of the 5-item Oral Health Impact Profile: OHIP5-Ar

**DOI:** 10.1186/s12955-018-1046-0

**Published:** 2018-11-20

**Authors:** Mohammed Nasser Alhajj, Esam Halboub, Nadia Khalifa, Abdullah G. Amran, Daniel R. Reissmann, Abbas G. Abdullah, Mounzer Assad, Abdulghani A. Al-Basmi, Fawaz A. Al-Ghabri

**Affiliations:** 1 Department of Prosthodontics, Faculty of Dentistry, Thamar University, Dhamar, Yemen; 2 Department of Maxillofacial Surgery and Diagnostic Sciences, College of Dentistry, Jazan University, Jazan, Saudi Arabia; 3 Department of Preventive and Restorative Dentistry, Faculty of Dental Medicine, University of Sharjah, Sharjah, United Arab Emirates; 4 Department of Periodontics, Faculty of Dentistry, Thamar University, Dhamar, Yemen; 5 Department of Prosthetic Dentistry, University Medical Center Hamburg-Eppendorf, Hamburg, Germany; 6 Department of Basic Sciences, Faculty of Dentistry, University of Khartoum, Khartoum, Sudan; 7 Department of Oral Surgery, Faculty of Dentistry, Tishreen University, Lattakia, Syria; 8 Private Dental Clinic, Dhamar, Yemen

**Keywords:** OHRQoL, OHIP5, Translation, Arabic version, Questionnaire

## Abstract

**Purpose:**

The aim of this study was to translate and validate an Arabic version of the 5-item Oral Health Impact Profile (OHIP).

**Methods:**

A total of 320 subjects (aged 18 years and above) were consecutively recruited from dental clinics. The self-administered OHIP5-Ar was distributed and the data were collected and analyzed. The dimensionality of the instrument was investigated using confirmatory factor analyses (CFA). Reliability was assessed as the instruments internal consistency using Cronbach’s alpha and test-retest-reliability using intraclass correlation coefficient (ICC). Convergent validity was tested by correlation between perceived global oral and general health questions with the latent factor (OHRQoL) using structural equation modelling analysis and with OHIP5-Ar total score using spearman’s correlation coefficient. Known-groups validity was tested among groups with known differences and sensitivity to change was also investigated after dental treatments.

**Results:**

The OHIP5-Ar was fitted well in the unidimensional model as indicated by the CFA with fit indices (RMSEA: 0.00, SRMR: 0.010, GFI: 0.998, TLI: 1). Cronbach’s alpha was 0.78 and the ICC agreement was 0.88. The validity tests indicated satisfactory validity of the instrument and the sensitivity to change of the instrument revealed significant change in the OHIP5-Ar total score after the provision of dental treatments (effect sizes: 0.55–1.49).

**Conclusion:**

The OHIP5-Ar showed satisfactory psychometric properties among Arabic-speaking population. This instrument is sensitive to the changes of oral health and can be used to measure the OHRQoL with one total score.

## Background

Quality of Life (QoL) is defined as individuals’ perception of their position in life with regard to the culture and value systems where they live, and in relation to their goals, expectations, standards and concerns [1]. It is not a simple concept; instead, it is a broad-ranging concept influenced in a complex way by the individuals’ physical health, psychological state, level of dependence, social relationships, and their relationships to salient features of the surrounding environment [1–3]. Primarily and directly oral health can affect the general health resulting in positive or negative impact on the individuals’ QoL, that is, they can eat, talk and conduct daily social activities without discomfort or embarrassment [1, 4]. When QoL is linked to health and disease, it is referred to as Health-Related Quality of Life (HRQoL). This term is useful to differentiate it from other QoL aspects. HRQoL is a multidimensional concept including physical, mental/emotional, and social functioning domains. It refers to the impact of health status on the individual’s quality of life [5–7].

A part of HRQoL is Oral Health-Related Quality of Life (OHRQoL), which is a multidimensional concept referring to patient’s perceptions when eating, sleeping, or engaging in social activities with respect to oral health [8–10]. The Oral Health Impact Profile (OHIP) is the most widely used instrument to measure OHRQoL [8]. Based on Locker’s conceptual model of oral health [11], Slade and Spencer developed and validated the original 49-item OHIP (OHIP49) [12]. It has been validated culturally and linguistically; its psychometric properties have been tested and it has been used in both cross-sectional and longitudinal studies [13–16]. The OHIP is usually used to evaluate the impact of oral disease on quality of life and to measure the outcomes of clinical interventions [17–19]. Despite the fact that it has been widely used and accepted, in addition to being comprehensive and precise when measuring OHRQoL, it is long and hence it is time-consuming, more prone to missing data, inconvenient, it costs more, and may causes problems especially to elderly respondents [20]. For the afore-mentioned reasons, several short forms of OHIP were created [20–23].

The shortest version of this instrument is the 5-item OHIP which, as its name implies, consists of 5 questions representing the four suggested dimensions: Oral Function, Orofacial Pain, Orofacial Appearance and Psychosocial Impact [24, 25]. It has only 10% of the original instrument items but can capture almost 90% of the information. This makes it a more accepted and attractive instrument for efficient OHRQoL assessment. It has been tested and validated in a Swedish general population [14], Japanese prosthodontic patients [26], Dutch temporomandibular dysfunction (TMD) patients [27], German general population and TMD patient [23], and a US adult general population [28]. So far, there has not been any information about the Arabic version of this ultra-short instrument.

The aim of this study, therefore, was to translate an Arabic version of the 5-item OHIP and to test its psychometric properties among an Arabic-speaking population.

## Materials and methods

### Translation of the OHIP5 into Arabic

Our version was translated from the English version published by Naik et al. [28] using a forward-backward approach [29]. A team of four fluent bilingual dentists were independently involved in this part of the study. The English version was translated to Arabic by two translators. The resulting versions were reviewed and integrated into one version. This version was backward translated to English by the other two translators who had no access to the English version. Similar to the forward translation, the resulting versions were reviewed and integrated into one version. Both forward and backward versions were revised and a consensus was achieved about discrepancies. Similar to the English version, responses to the OHIP5-Ar questions were made on a 5-point Likert scale (0 = never; 1 = hardly ever; 2 = occasionally; 3 = fairly often; and 4 = very often).

A comparison regarding content and wording between the translated OHIP5 items and the corresponding items in the longer forms of the Arabic versions [30, 31] was performed to make sure that they are equivalent. The initially developed Arabic-language version was piloted on a convenience sample. Thirty subjects were involved and their comments were received and addressed. The final Arabic-language version of the instrument (OHIP5-Ar) was then finalized and prepared for the main study (Appendix). The piloted subjects were not involved in the main study.

### Patient involvement and data collection

The study comprised 320 subjects aged 18 years and older. This number of participants exceeded the recommended subject to item ratio (2–20 subjects per item) which is needed to perform factor analyses [32–34]. They were recruited consecutively from patients and their accompanying persons who attended one specialist and two general dental clinics in Dhamar city, Yemen. The protocol of this study was approved by Ethics Committee of the Faculty of Dentistry, Thamar University (Ref: 2016009). Before commencing the study, its aims were explained to the participants and they were asked to give their informed consent. In addition to the OHIP5-Ar questions, the questionnaire included sociodemographic questions and two global questions related to perceived oral and general health status reported on an ordinal 5-point scale (‘poor’, ‘fair’, ‘good’, ‘very good’, and ‘excellent’) [31, 35, 36]. All participants were subjected to clinical assessment which included the periodontal status and number and location of missing teeth (anterior or posterior). For evaluation of gingival inflammation and periodontal attachment loss, the diagnostic criteria of the periodontal index were applied [37]. Number of present teeth were classified as < 20 teeth or ≥ 20 teeth [38, 39]. One specialist in periodontics and two other well-trained authors performed the examination.

### Statistical analysis

#### Reliability

The reliability of the OHIP5-Ar was assessed using internal consistency and test-retest reliability. Cronbach’s alpha and inter-item correlation were used for internal consistency. Values of Cronbach’s alpha ≥0.70 were considered satisfactory [40], and for inter-item correlation, values > 0.20 were considered acceptable [41]. Based in a one-way random-effects ANOVA, the intraclass correlation coefficient (ICC) were calculated to determine test-retest reliability. A convenience sample of thirty subjects were selected for this test with a 2 weeks interval between the two assessments and no treatment performed in between. To ensure that, this group was selected from co-patients. However, only 26 subjects were available for second assessment. Values of ICC of > 0.80 indicate excellent agreement, good agreement with values from 0.61 to 0.80, moderate agreement with values from 0.41 to 0.60, and poor agreement with values < 0.40 [42]. Subjects recruited for the reliability test were not included in the main study.

#### Validity

Since OHIP5 has one dimension, one OHIP summary score is sufficient to express the individual’s OHRQoL [23]. With this regard, construct validity and dimensionality of the OHIP5-Ar were assessed by confirmatory factor analysis (CFA). All items of the OHIP5-Ar were loaded to one latent factor representing OHRQoL. The model fit was evaluated using a set of indices including: good of fitness index (GFI), standardized root mean square residual (SRMR), root mean square error of approximation (RMSEA), incremental fit index (IFI) which is considered analogous to R^2^, and Tucker-Lewis index (TLI). The following values were suggested as guidelines for model fit: GFI ≥0.95, SRMR ≤0.08, RMSEA ≤0.06, IFI ≥0.95 and TLI ≥0.95 [43–46]. The latent factor was considered to have a mean of 0 and a variance of 1 for identification purpose. To improve the model fit and to avoid overestimation or underestimation of the model [47–51], correlation between measurements errors (e1 and e2, e1 and e5, e2 and e5) were allowed.

Convergent validity was assessed by Spearmen’s rank correlation between summary score of the OHIP5-Ar and both global questions of perceived oral and general health status. The global general health question was included considering that this question implicitly includes the oral health status as a part of the general health. Moreover, it is well known that there is a considerable correlation between perceived oral health and general health [52–54]. The structural equation modelling analysis (SEM) was also performed using one latent factor representing OHRQoL and was correlated with global oral and general health questions. The above mentioned indices and guidelines were followed for model fit.

For known-groups validity, the differences between OHIP5-Ar summary scores of different groups expected to have different OHRQoL impairment were assessed. We compared OHIP5-Ar summary scores between subjects who had ≥20 teeth and those who had < 20 teeth [55, 56], subjects with periodontal problems and those with healthy periodontium [57, 58], subjects with age ≤ 40 years and those with age > 40 years [55, 59], subjects with anterior missing teeth (affecting esthetic appearance) and those with posterior missing teeth [56, 60–63], and subjects with different educational status would have different values of OHRQoL [64, 65].

#### Sensitivity to change

It was hypothesized that responses of patients to OHIP5-Ar and thus OHRQoL will change after the provision of dental treatments. Thirty patients with different treatment needs were selected to assess sensitivity to change of the OHIP5-Ar. They were asked to fill the questionnaire at the first visit before starting any dental treatment and to refill it again after a recall period of 10 days to 1 month. The dental treatments included: gingivectomy (*n* = 5), anterior fixed partial denture (*n* = 10), posterior fixed partial denture (n = 10), and complete denture prosthesis (n = 5). The effect size (Cohen’s d) was calculated using the following equation: (mean of baseline OHIP score ─ mean of follow-up OHIP score) / standard deviation of the baseline OHIP score [66]. According to Cohen, an effect size of d = 0.2 is considered to be small, 0.5 is medium, and 0.8 is large.

Test of normality revealed non-normal distribution of the data so that non-parametric tests were used as appropriate. Statistical tests and path analysis were performed using statistical software (IBM SPSS Statistics v22 and AMOS v23; IBM Corp) (α = 0.05 for all tests).

## Results

### Subjects characteristics

The majority of participants were female (73.1%) and married (73.4%) (Table 1). Mean age was 32.0 ± 13.2 years. Educational status varied among the participants (more proportions were in secondary and university levels). Most participants had ≥20 teeth (83.1%), missing posterior missing teeth (74.4%), and periodontal problems (73.1%). Regarding the global questions of oral and general health, most participants reported them as good.

**Table 1:** Characteristics of the study sample

	N	%
Gender		
Male	86	26.9
Female	234	73.1
Marital status		
Single	85	26.6
Married	235	73.4
Age group (years)		
< 20	41	12.8
20–39	128	40.0
30–39	70	21.9
40–49	32	10.0
50–59	31	9.7
≥ 60	18	5.6
Education		
Illiterate	49	15.3
Primary	58	18.1
Preparatory	60	18.8
Secondary	75	23.4
University	72	22.5
Above	6	1.9
Number of remaining teeth		
≥ 20 teeth	266	83.1
< 20 teeth	54	16.9
Location of missing teeth		
Anterior	82	25.6
posterior	238	74.4
Periodontal status		
Healthy	86	26.9
Unhealthy	234	73.1
Perceived general health		
Excellent	55	17.2
Very good	92	28.8
Good	122	38.1
Fair	42	13.1
Poor	9	2.8
Perceived oral health		
Excellent	17	5.3
Very good	43	13.4
Good	111	34.7
Fair	92	28.8
Poor	57	17.8

### Reliability

Cronbach’s alpha of the OHIP5-Ar was 0.78 ranging from 0.71 to 0.78 when single items were deleted (Table 2), indicating satisfactory internal consistency for the entire instrument. Inter-item correlation between each pair of two items ranged from 0.33 to 0.65. All inter-item correlations were found to be above the recommended value of 0.20 (Table 3).

**Table 2:** Means, SD, floor and ceiling effect, and Cronbach’s alpha of OHIP5-Ar items

	Mean ± SD	Range	Floor and ceiling effect		Cronbach’s Alpha if Item Deleted
			% of value 0	% of value 4	
Difficulty chewing	1.67 ± 1.43	0–4	33.4	12.2	0.72
Painful aching	1.54 ± 1.29	0–4	30.6	7.5	0.71
Uncomfortable about appearance	1.56 ± 1.43	0–4	35.6	12.2	0.78
Less flavor in food	1.05 ± 1.23	0–4	50.0	5.3	0.73
Difficulty doing usual jobs	0.90 ± 1.16	0–4	53.4	3.8	0.76

*SD* standard deviation; Total Cronbach’s alpha = 0.78

**Table 3:** Inter-Item Correlation Matrix of OHIP5-Ar

	Difficulty chewing	Painful aching	Uncomfortable about appearance	Less flavor in food	Difficulty doing usual jobs
Difficulty chewing	1.00				
Painful aching	0.65	1.00			
Uncomfortable about appearance	0.33	0.40	1.00		
Less flavor in food	0.47	0.48	0.34	1.00	
Difficulty doing usual jobs	0.37	0.34	0.35	0.47	1.00

With respect to test-retest-reliability, the ICC of 0.88 for the OHIP5-Ar summary score and 0.74 to 0.97 for the single items indicated excellent agreement between the two assessments. Using Paired t test, all differences between both readings were minimum and insignificant (P >0.05) (Table 4).

**Table 4:** Test-retest of reliability and differences between OHIP5-Ar items readings

OHIP5	ICC	95% CI	*P*	Mean difference ± SD^a^	*P*
ALL	0.88	0.80-0.94	< 0.001		
Difficulty chewing	0.78	0.50–0.90	< 0.001	0.31 ± 0.97	0.118
Painful aching	0.79	0.54–0.91	< 0.001	0.04 ± 1.04	0.852
Uncomfortable about appearance	0.97	0.93–0.99	< 0.001	0.00 ± 0.49	1.000
Less flavor in food	0.74	0.42–0.88	0.001	0.19 ± 0.63	0.134
Difficulty doing usual jobs	0.84	0.65–0.93	< 0.001	0.12 ± 0.91	0.523
Summary core				0.65 ± 1.98	0.104

*ICC* Intraclass Correlation Coefficient, *SD* standard deviation; *P* value is significant at 0.05 level

^a^Paired sample t-test

### Validity

#### Construct validity and dimensionality

**Fig. 1:**
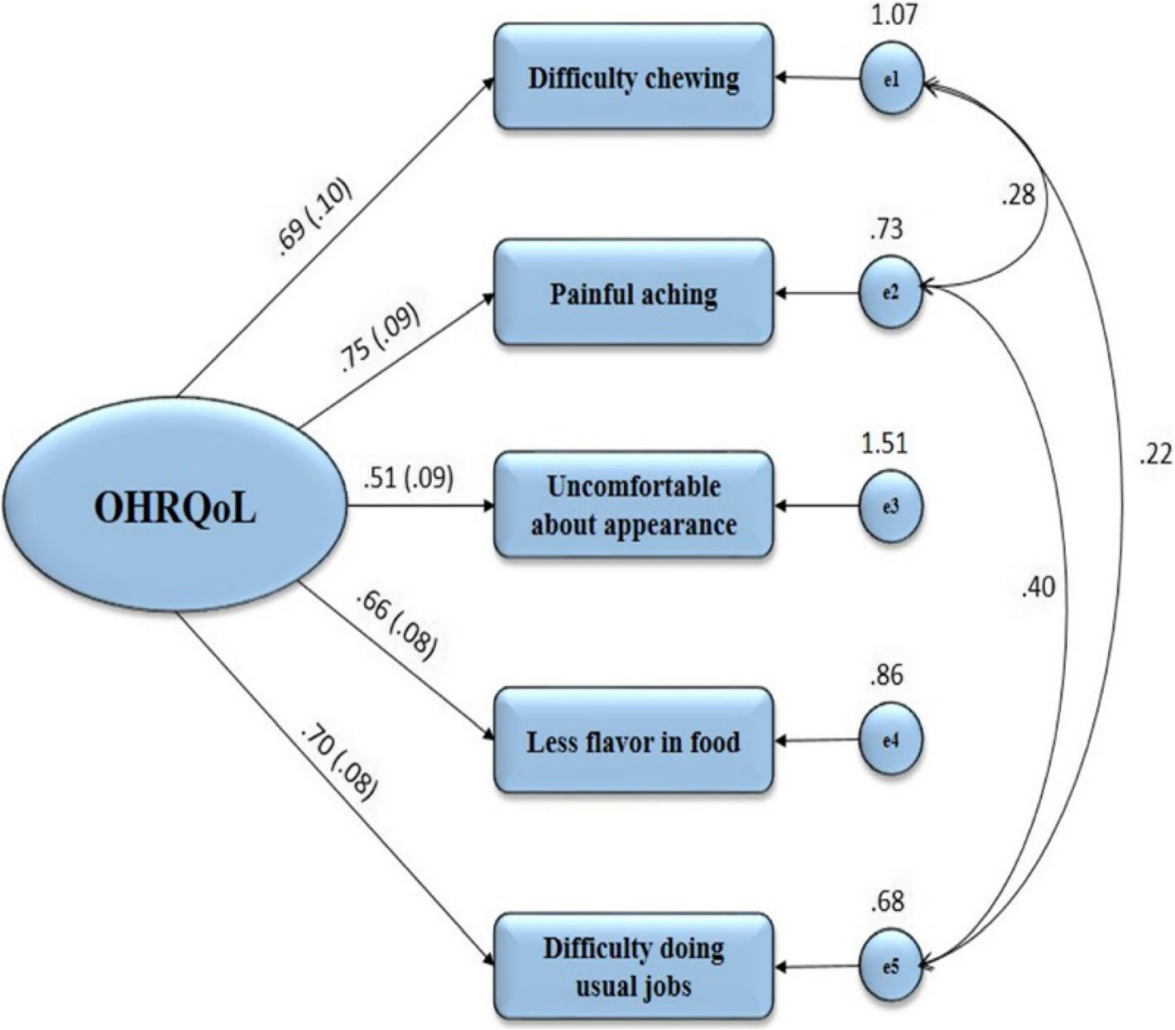
Confirmatory factor analysis model of the oral health-related quality of life (OHRQoL) measured with the five-item OHIP. The latent factor (OHRQoL) is presented in oval and the rectangles represents the measured indicators of the latent factor with their variance. The uni-directional lines arising from the latent factor refer to the factor loadings of the indicators (with their standard errors). Numbers on these lines refer to the standardized values

The data fit the unidimensional model well (GFI: 0.998, SRMR: 0.010, RMSEA: 0.00, IFI: 1, TLI: 1). All these indices indicate excellent fit of the model, unidimensionality, and that the OHIP5-Ar structure can be modeled well by one latent factor model. Standardized estimates ranged from 0.51 to 0.75 with small standard errors (0.05–0.10), indicating that all items were strong indicators of the latent OHRQoL factor (Fig. 1).

#### Convergent validity

The summary score of the OHIP5-Ar was highly and statistically significantly correlated to the single-item measure of perceived oral health (*r* = 0.44, *P* <  0.001) (Table 5). Similarly, a statistically significant correlation was found between summary score of the OHIP5-Ar and perceived general health (*r* = 0.17, *P* <  0.001). This correlation, however, was lower than that for perceived oral health, as expected. Moreover, age (as continuous variable) showed highly statistically correlation (*r* = 0.21; *P* <  0.001) with the OHIP5-Ar indicating increase in the self-rating scores with age progress.

**Table 5:** Convergent validity of OHIP5-Ar

		OHIP5	Correlation Coefficient		
		Mean ± SD	*rho* ^a^	95% CI	P
Perceived oral health (*n* = 320)			0.44	(0.35–0.53)	< 0.001
	Excellent (*n* = 17)	0.65 ± 0.60			
	Very good (*n* = 43)	0.83 ± 0.68			
	Good (*n* = 111)	1.14 ± 0.88			
	Fair (*n* = 92)	1.41 ± 0.81			
	Poor (*n* = 57)	2.23 ± 0.97			
Perceived general health (n = 320)			0.17	(0.06–0.27)	< 0.001
	Excellent (*n* = 55)	1.14 ± 0.98			
	Very good (*n* = 92)	1.15 ± 0.86			
	Good (*n* = 122)	1.51 ± 0.94			
	Fair (*n* = 42)	1.53 ± 1.05			
	Poor (*n* = 9)	1.44 ± 1.05			

*ICC* Intraclass Correlation Coefficient, *SD* standard deviation; *P* value is significant at 0.05 level

^a^Spearman’s correlation coefficient test

**Fig. 2:**
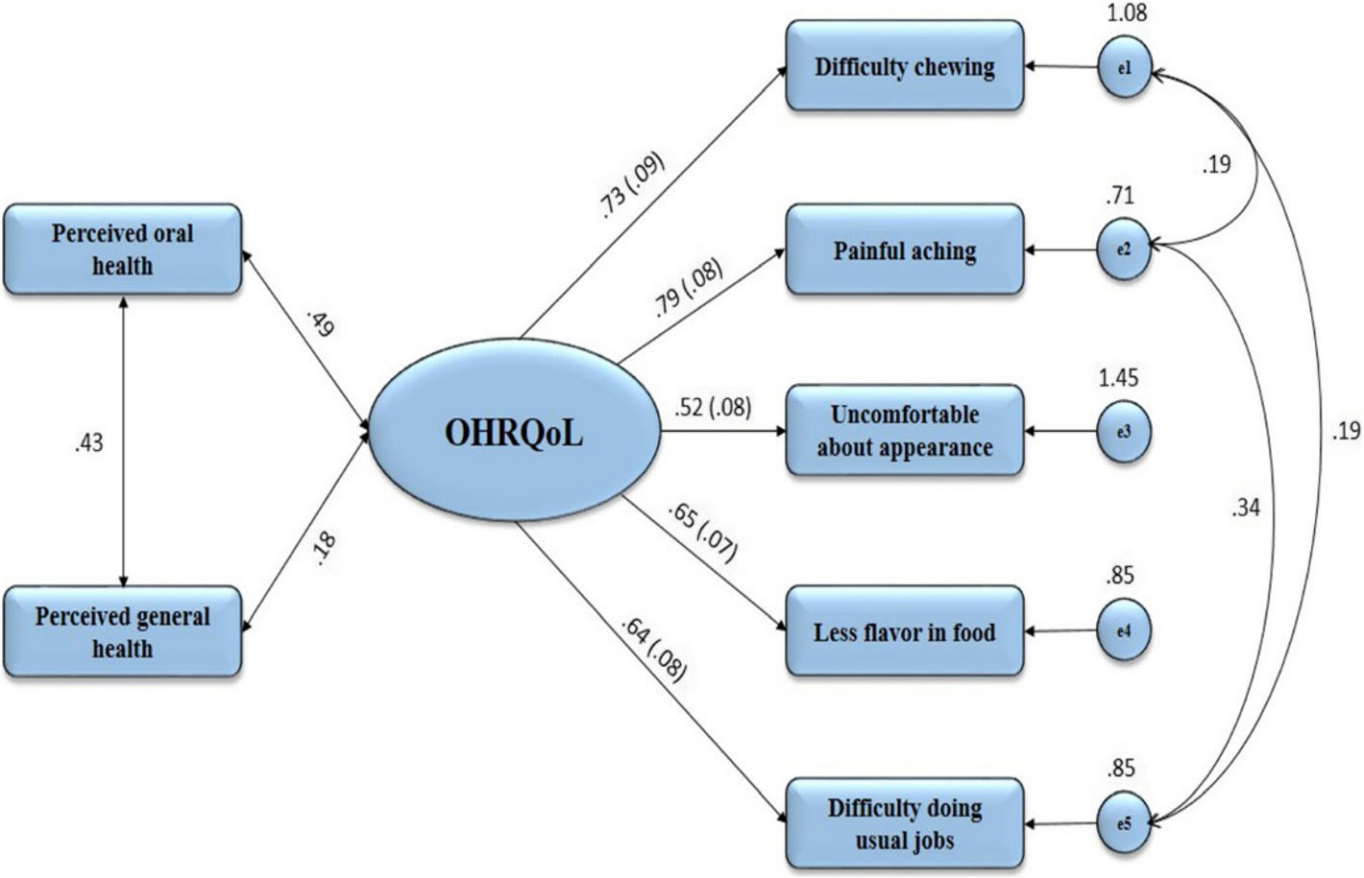
Structural equation modelling (SEM) analysis for the correlation between perceived global oral and general health question and the latent factor (OHRQoL). The rectangles on the left represent perceived global oral and general health questions while the rectangles on the right represent the measured indicators of the latent factor with their variance. The uni-directional lines arising from the latent factor refer to the factor loadings of the indicators (with their standard errors). Number on these lines refer to the standardized values. The bidirectional lines refer to the correlation between perceived global oral and general health question and the latent factor (OHRQoL)

All items in the structural equation model were combined to one common (general) factor representing OHRQoL. Data fit the model quite well (GFI: 0.977, SRMR: 0.043, RMSEA: 0.075, IFI: 0.970, TLI: 0.937), even though the value for RMSEA was somewhat higher and the value for TLI somewhat lower than the threshold for excellent fit. The correlations between the latent factor (OHRQoL) and both perceived oral and general health items were substantial (*r* = 0.49 and *r* = 0.18, respectively). These values were close to those resulting from Spearman’s correlation test confirming that the OHIP5-Ar can be modeled by a latent factor (Fig. 2).

#### Known-groups validity

The analyses of the potentially different groups (missing teeth, location of the missing teeth, periodontal status, age, and educational status) revealed highly statistically significant differences indicating sufficient known-groups validity of the OHIP5-Ar (Table 6). As expected, patients with less teeth, missing anterior teeth or periodontal problems had more problems and thus lower OHRQoL than the corresponding patient groups.

**Table 6:** Known-groups validity of OHIP5-Ar

	N	Mean ± SD	*P*
Missing teeth^a^			
≥20 teeth	266	1.18 ± 0.92	< 0.001
< 20 teeth	54	2.15 ± 0.70	
Location of missing^a^			
Anterior	82	1.87 ± 0.96	< 0.001
Posterior	238	1.16 ± 0.89	
Periodontal status^a^			
Unhealthy	234	1.43 ± 0.96	0.006
Healthy	86	1.11 ± 0.92	
Age^a^			
≤ 40	251	1.25 ± 0.94	< 0.001
> 40	69	1.69 ± 0.93	
Education level^b^			
Illiterate	49	2.02 ± 0.89	< 0.001
Primary	58	1.40 ± 0.95	
Preparatory	60	1.24 ± 0.98	
Secondary	75	1.18 ± 0.98	
University	72	1.13 ± 0.77	
Postgraduate	6	0.93 ± 0.78	

*SD* standard deviation, *P* value is significant at 0.05 level

^a^Mann-Whitney test; ^b^Kruskal-Wallis test

### Sensitivity to change

Pre-treatment summary scores of the OHIP5-Ar increased significantly after the provision of different dental treatments. The mean difference of the OHIP5-Ar summary scores between pre- and post-treatment was 6.2 ± 4.9 (effect size: 1.31, *P* <  0.001. Effect sizes of the individual items ranged from 0.55 to 1.49 indicating moderate to large effects of the intra-individual differences between the two assessments (Table 7). Moreover, all effect sizes exceeded the threshold of 0.5 for a clinically relevant change [67].

**Table 7:** Sensitivity to changes of OHIP5-Ar (pre- and post-treatment)

OHIP5	Pre-treatment	Post-treatment	Mean difference	Effect size	*P* ^a^
Difficulty chewing	2.07 ± 1.46	0.87 ± 1.07	1.20 ± 1.45	0.82	0.001
Painful aching	1.80 ± 1.16	0.60 ± 0.93	1.20 ± 1.30	1.04	< 0.001
Uncomfortable about appearance	2.50 ± 1.46	0.33 ± 0.66	2.17 ± 1.66	1.49	< 0.001
Less flavor in food	1.33 ± 1.30	0.33 ± 0.71	1.00 ± 1.08	0.77	< 0.001
Difficulty doing usual jobs	0.90 ± 1.21	0.23 ± 0.63	0.67 ± 1.06	0.55	0.002
Summary	8.60 ± 4.76	2.37 ± 3.19	6.23 ± 4.88	1.31	< 0.001

*P* value is significant at 0.05 level; ^a^Wilcoxon Signed Ranks test

## Discussion

This is the first study assessing the psychometric properties of the OHIP5 in its Arabic version. Findings indicate satisfactory validity of the instrument suggesting its use as one total score to assess the OHRQoL in Arabic-speaking population. Our findings are well comparable to those from other studies on the 5-item OHIP. The dimensionality test of OHIP5-Ar revealed a one-dimensional instrument containing five items, each referring to at least one of the four suggested dimensions of the longer versions. Similar results were found in the English [28] and German [23] versions of the OHIP5. The internal consistency of the OHIP5-Ar was 0.78 indicating satisfactory construct validity of the instrument. This value is higher than that of the Dutch version [27] which was 0.67, lower than that of the Japanese version [26] which was 0.81, but it is close to the German [23], Swedish [14], and English [28] versions (0.76, 0.77, and 0.75, respectively). The average inter-item correlation was 0.42 which is similar to the Swedish version (0.41) [14]. The ICC of 0.88 was somewhat higher than that of the Dutch [27], German [23], and Japanese [26] versions. But differences were not considered relevant.

Correlation between summary score of the OHIP5-Ar and global perceived oral health question was close to that resulted from the latent variable analysis, indicating fit of the model for the target population. Similarly, the correlations between the summary score of the OHIP5-Ar and global general health question and that resulted from SEM analysis were close to each other. This confirms that the individual items of the OHIP5-Ar are able to measure the construct OHRQoL. The correlation between the OHIP5-Ar and global oral health is similar to those observed in the German (*r* = 0.50) [23] and Japanese (*r* = 0.48) [26] versions and higher than that found in the English version (*r* = 0.30) [28]. The SEM-based correlation, however, is close to that of the English version (*r* = 0.46) [28]. The OHIP5-Ar could clearly and significantly differentiate between different groups that have potential differences. It is well-known in the dental literature [56] that patients with more remaining teeth (≥20 teeth) have better oral function than those with fewer remaining teeth (< 20 teeth), and this will be primarily mirrored in their OHRQoL. In our study, patients with ≥20 teeth had lower values of the OHIP5-Ar score (better oral health) than those who had < 20 teeth. Esthetic appearance can also affect the individual’s performance and thus the quality of life [61–63]. The OHIP5-Ar includes a question (Q3) related to esthetic which represents the orofacial appearance domain in the longer versions. Accordingly, patients who presented with missing posterior teeth reported better oral health than patients with missing anterior teeth. Similarly, patients with unhealthy periodontium reported worse OHRQoL than those did with healthy periodontium. Pain, bleeding, tooth mobility, calculus deposition, and gum recession were some of the clinical features of the diseased periodontium which reflected negatively on the patient’s response. Increasing age is considered one of the contributing factors for poor oral health. This was obvious with our sample where patients ≤40 years had lower values (positive response) of the OHIP5-Ar than those with age > 40 years. Different values of oral health were observed among subjects with different educational levels. This was not surprising as an educated subject is likely to know more about oral health and care.

Sensitivity to change of the OHIP5-Ar was clearly confirmed in our sample. The effect of dental treatments on patients’ response was moderate to large. This effect is similar to that obtained in the German [23] and Japanese [26] versions. The relevant sample in our study received different types of dental treatments which is in contrast with the previous versions of OHIP5 where patients received the same type of dental treatment (provision of new or remake a denture in the Japanese version and patient’s with TMD pain in the German version). The ability of OHIP5-Ar to predict the changes of different types of dental treatment has been proven in our study. It makes this instrument more suitable to be used in everyday dental practice. For assessing sensitivity to change, the time between intervention and recall assessment ranged from 10 days to 1 month. Even though the OHIP usually has a recall period of 1 month [23, 26], i.e., respondents are asked what they have experienced during the previous month and requiring at least 1 month between an intervention and the follow-up assessment to capture the entire treatment effect, changes in OHIP5-Ar summary scores were statistically significant and of moderate to large effect size. This also suggests that a shorter recall period (7 days) as suggested by Waller et al. [68], where patients are asked about their experiences during the previous week, could also be applied for the OHIP5-Ar. However, we believe that the ability of OHIP to detect the changes during this short period depends on the type of treatment itself. For example, adaptation to 3-unit fixed partial denture will take shorter time than adaptation to lower complete denture, and this will be definitely mirrored in the patient’s response.

In comparison with the longer Arabic versions of this instrument which were validated by Al-Jundi et al. (OHIP49-Ar) [30] and Khalifa et al. (OHIP14-Ar), [31] the internal consistency of our version is higher than that of the OHIP49-Ar version (0.74), [30] and similar to that of the OHIP14-Ar (0.80). [31] The inter-item correlation and ICC agreement are also higher in our version. However, the correlation between OHIP5-Ar and both global oral and general health questions are lower than those found in the OHIP49-Ar version. [30] The OHIP14-Ar version [31] also reported highly significant correlations between these variables but no coefficients (*r*) were mentioned. The higher correlation coefficients in the OHIP49-Ar version might be related to the number of questions (49 questions compared with 5 question) which can influence the relationships between these variables.

Dental status as well as dental treatment can greatly affect the individual’s QoL. Dental caries [69], teeth missing [56], gum disease [70], aphthous ulcers [71], and temporomandibular disorders [72] are some examples of such pathologies that affect the QoL. Moreover, dental anxiety can also affect the individual perception of QoL [73]. On the other hand, many or almost all dental treatments can significantly improve the individual’s QoL [74–77]. The current study has strengths and limitations. We used subjective measures of perceived oral and general health and objective measures of physical oral health status for validity assessments, what is considered a strength of the study. Furthermore, we applied sophisticated statistical analyses, i.e., SEM analysis and latent factor model. The sample size was sufficiently large to unambiguously prove reliability, validity, and sensitivity to change of the OHIP5-Ar. Our sample was recruited from specialist and general dental clinics attenders. A longer version of OHIP (OHIP14) was also validated among general dental clinics attenders in Scotland [78]. Although our study population could be considered a representative for dental patients, a community-based study with large sample size is recommended to validate the instrument among the general population. We didn’t correlate the OHIP5-Ar to the long Arabic version (OHIP49-Ar). Since prior studies have reported a high correlation between short and long versions [14, 26, 28], therefore this study did not focus on highlighting associations on this subject. Only one component (missing teeth) of the DMFT index was assessed, further studies could include this index to further validate the instrument.

## Conclusion

The Arabic-language version of the OHIP5 is a valid and reliable instrument to assess OHRQoL in an Arabic-speaking population. Due to sufficient psychometric properties, low burdens, and easy applicability it can be recommended to be used in dental practice and for research purposes as well.

## Figures and Tables

**Table 8:**
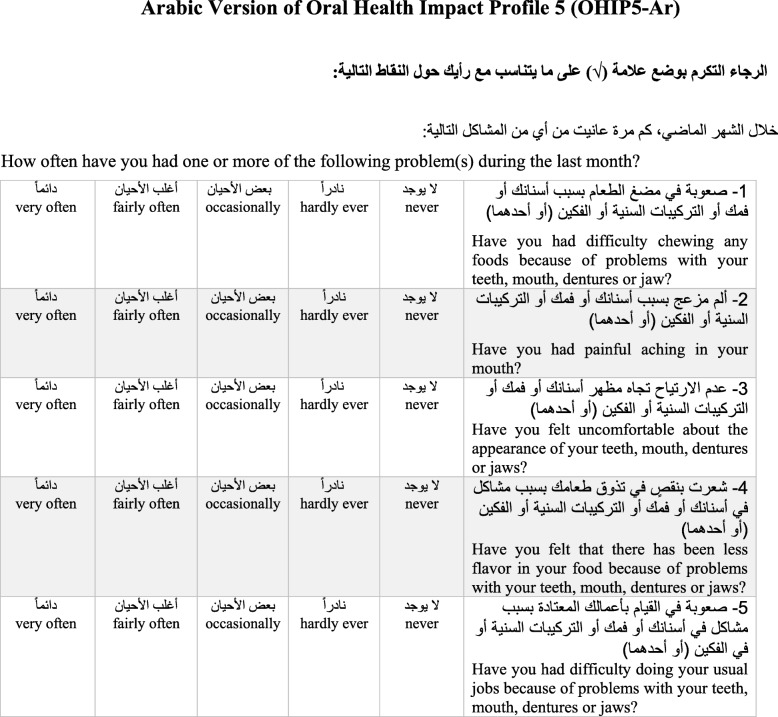
Arabic Version of Oral Health Impact Profile 5 (OHIP5-Ar)

## Abbreviations

CFA: Confirmatory Factor Analysis
GFI: Goodness of Fit Index
ICC: Intraclass Correlation Coefficient
IFI: Incremental Fit Index
OHIP: Oral Health Impact Profile
OHRQoL: Oral Health-Related Quality of Life
QoL: Quality of Life
RMSEA: Root Mean Square Error of Approximation
SEM: Structural Equation Modelling
SRMR: Standardized Root Mean Square Residual
TLI: Tucker-Lewis Index
TMD: Temporomandibular Disorder
